# C - reactive protein to albumin ratio as predictor of severity and outcome in acute pancreatitis

**DOI:** 10.6026/973206300223521

**Published:** 2026-06-30

**Authors:** Umesh Pratap Singh, Manzar Hussain Usmani, Mukund Pandey, Pooja Patel

**Affiliations:** 1 Department of General Medicine, Shyam Shah Medical College and Associated Sanjay Gandhi Memorial Hospital, Rewa, Madhya Pradesh, India; 2 Department of Gastroenterology, Shyam Shah Medical College and Associated Sanjay Gandhi Memorial Hospital, Rewa, Madhya Pradesh, India; 3 Department of General Surgery, Shyam Shah Medical College and Associated Sanjay Gandhi Memorial Hospital, Rewa, Madhya Pradesh, India

**Keywords:** Acute pancreatitis (AP), serum albumin, C - reactive protein (CRP)/albumin ratio

## Abstract

Early and accurate prediction of severity in acute pancreatitis remains a major clinical challenge, particularly in resource-limited settings. Hence, this cross-sectional study evaluated 100 patients to assess the prognostic value of the CRP/Albumin ratio (CAR) in comparison with BISAP and other parameters. Disease severity was mild in 40%, moderate in 30% and severe in 30%, with ICU admission in 23% and overall mortality of 5%, increasing with severity. Non-survivors had significantly higher age (62.6 ± 7.09 vs 40.41 ± 13.66 years), urea (78.72 ± 66.08 vs 24.09 ± 13.99 mg/dL), creatinine (2.69 ± 1.68 vs 0.84 ± 0.43 mg/dL), CRP (182.61 ± 141.55 vs 75.98 ± 82.76 mg/L), CAR (107.18 ± 106.30 vs 28.09 ± 38.10) and BISAP scores (4.6 ± 0.55 vs 0.85 ± 0.99), along with lower albumin levels (2.11 ± 0.87 vs 3.20 ± 0.98 g/dL). Thus, BISAP showed the highest predictive accuracy (AUC = 1.000), followed by creatinine (0.985), age (0.952) and CAR (0.930).

## Background:

Acute pancreatitis (AP) is a potentially life-threatening condition characterized by pancreatic inflammation [1]. It represents a significant clinical problem with varied severities, ranging from mild to severe disease [2]. Acute pancreatitis can lead to multi-organ failure and death, making accurate prediction of its course and outcome critical for clinicians [3]. The ability to predict severity early enables timely interventions that reduce complications and improve outcomes [4]. Various biomarkers have been investigated as predictors of severity [5]. C - reactive protein (CRP) is an acute-phase reactant produced by the liver in response to inflammation [6]. Its levels rise quickly in acute illness, reflecting inflammation intensity [7]. Elevated CRP has been associated with severity in various inflammatory conditions [8]. Albumin is synthesized by the liver to maintain oncotic pressure and transport substances [9]. Low albumin levels occur in systemic inflammation, liver dysfunction, or malnutrition [10]. In acute pancreatitis, hypoalbuminemia is common and linked to poor prognosis [11]. The CRP/Albumin ratio combines CRP, reflecting inflammation, with albumin, representing nutritional and functional status [122]. This ratio has shown promise as an early prognostic tool in acute pancreatitis [13]. Clinical applications include identifying high- risk patients requiring ICU monitoring [14]. Such patients may need aggressive interventions including nutritional support [15]. The ratio helps stratify patients for surgical intervention when indicated [16]. Additionally, the CRP/Albumin ratio aids in selecting patients for clinical trials [17]. Despite the potential of the CRP/Albumin ratio as a prognostic marker, limited data exists on its predictive accuracy in our population. Therefore, it is of interest to evaluate the role of the CRP/Albumin ratio in predicting severity and outcomes in patients with acute pancreatitis.

## Materials and Methods:

## Study design and setting:

This was an observational, cross-sectional study conducted in the Department of General Medicine at Shyam Shah Medical College and Sanjay Gandhi Memorial Hospital, Rewa, Madhya Pradesh. The study period spanned January 2024 to December 2024.

## Study population: 

A total of 100 patients diagnosed with acute pancreatitis (AP) were enrolled. The diagnosis was established based on clinical features, biochemical markers (serum amylase/lipase >3 times the upper limit of normal) and/or radiological evidence (CT/MRI/ultrasound findings consistent with AP), as per standard guidelines.

## Inclusion criteria:

Patients aged 18 years or older with a confirmed diagnosis of acute pancreatitis and admission within 48 hours of symptom onset were included in the study.

## Exclusion criteria:

Patients with a history of chronic pancreatitis or pancreatic malignancy, known chronic liver disease or nephrotic syndrome, those receiving immunosuppressive therapy or diagnosed with systemic inflammatory diseases and pregnant patients were excluded from the study.

## Data collection:

On admission, each patient underwent a comprehensive clinical evaluation, including detailed history taking to document demographic characteristics, comorbidities and substance use (alcohol and tobacco). Within the first 24 hours of hospitalization, blood samples were collected to assess laboratory parameters: complete blood count (CBC), renal function tests (serum urea and creatinine) and liver function tests including serum albumin, C - reactive protein (CRP) and serum amylase and lipase levels. The CRP/Albumin Ratio (CAR) and the BISAP score (Bedside Index for Severity in Acute Pancreatitis) were calculated at the time of admission. In addition, all patients underwent radiological imaging primarily abdominal ultrasonography for initial evaluation and contrast-enhanced computed tomography (CECT) when clinically indicated to assess pancreatic morphology and complications and to classify disease severity based on the Revised Atlanta Classification.

## Classification of severity:

Disease severity was assessed according to the Revised Atlanta Classification (2012). Mild acute pancreatitis was defined by the absence of organ failure and local or systemic complications. Moderately severe acute pancreatitis was characterized by transient organ failure lasting less than 48 hours and the presence of local or systemic complications without persistent organ failure. Severe acute pancreatitis was defined by persistent organ failure lasting more than 48 hours involving one or more organ systems.

## Outcome measures:

The primary outcome of the study was in-hospital mortality among patients diagnosed with acute pancreatitis. Secondary outcomes included the length of hospital stay, the severity of disease based on the Revised Atlanta Classification (2012) and BISAP (Bedside Index for Severity in Acute Pancreatitis) score and the association of clinical and biochemical markers with outcomes such as organ dysfunction, inflammatory burden and need for intensive care. Biochemical parameters like CRP, serum albumin, urea, creatinine and the CRP/Albumin Ratio (CAR) were evaluated at 72 hours of admission and correlated with disease severity and mortality. The BISAP score was used as a bedside prognostic tool, calculated within the first 24 hours of admission.

## Data analysis:

Data were analyzed using SPSS (version 30) and/or Python/R. Continuous variables were summarized as mean ± standard deviation (SD), while categorical variables were expressed as counts and percentages. Group comparisons (e.g., survivors (discharged) vs. non-survivors (death), BISAP <2 vs. ≥2) were made using the student's t-test for continuous variables and the Chi-square or Fisher's exact test for categorical variables. Variables with p < 0.05 in univariate analysis were included in a multivariate logistic regression model to identify independent predictors of mortality. Results were expressed as odds ratios (OR) with 95% confidence intervals (CI). Receiver Operating Characteristic (ROC) curves was constructed to evaluate the diagnostic performance of key predictors. The area under the ROC curve (AUC) was calculated to determine accuracy. As presented in Table 1, the study included 100 patients diagnosed with acute pancreatitis. The mean age of the cohort was 41.86 ± 14.67 years, with the majority falling within the 41-50 years age group (28%), followed by 31-40 years (23%) and 51-60 years (20%), indicating that acute pancreatitis predominantly affects middle-aged individuals. Patients younger than 20 years constituted only 4%, while 9% were over 60 years of age. A clear male predominance was observed, with 64% of cases occurring in males and 36% in females. In terms of socioeconomic status, the majority of patients (72%) belonged to the lower socioeconomic class, followed by 26% from the middle class and only 2% from the upper class, suggesting a higher prevalence among economically disadvantaged populations. Additionally, 57% of the patients were from rural backgrounds, while 43% resided in urban areas, reflecting a slightly higher disease burden in rural settings.

## Results:

The etiological distribution revealed that gallstones were the leading cause of acute pancreatitis, accounting for 35% of cases. This was followed by alcohol-related pancreatitis (25%), combined alcohol and tobacco use (15%), hypertriglyceridemia (10%), idiopathic cases (10%) and drug-induced pancreatitis (5%). In terms of clinical outcome, 95% of patients were successfully discharged, while 5% succumbed to the disease, indicating a relatively favorable overall prognosis in this cohort. As shown in Table 2, all patients (100%) presented with abdominal pain, affirming its role as the hallmark symptom of acute pancreatitis. Nausea and vomiting were also highly prevalent, reported in 81% of cases. Other gastrointestinal manifestations included abdominal distension in 25% of patients and fever in 22%, reflecting systemic inflammatory response in a significant proportion. Less frequent but clinically important findings included icterus (jaundice) in 12%, suggesting possible biliary obstruction or hepatic involvement. Dyspnoea was reported in 10% of cases, possibly indicative of systemic inflammatory response syndrome (SIRS) or pleural effusion. More severe presentations were less common but notable, with altered sensorium in 4% and shock at admission observed in 3%, highlighting cases with potentially life-threatening complications at presentation. As presented in Table 3, the severity of acute pancreatitis, as classified by the Revised Atlanta Classification, demonstrated a strong and graded association with clinical outcomes, complication rates and mortality. Among the study cohort (N = 100), 40% of patients were categorized as having mild disease, 30% as moderate and 30% as severe. The mean hospital stays increased significantly across severity categories, from 5.2 ± 1.1 days in mild cases to 8.4 ± 2.0 days in moderate and 13.1 ± 3.5 days in severe cases (p < 0.0001). ICU admission was required in 23% of patients overall, with a markedly higher proportion in the severe group (66.7%) compared to the moderate group (10%) and none in the mild category (p < 0.0001). Similarly, the need for mechanical ventilation increased with disease severity, with 36.7% of severe and 6.7% of moderate patients requiring ventilation and none among mild patients (p < 0.0001). Mortality was confined to patients with moderate and severe pancreatitis, with one death in the moderate group (3.3%) and four deaths in the severe group (13.3%) (p = 0.0357). All patients with mild pancreatitis survived and were discharged successfully (100%), compared to 96.7% in the moderate and 86.7% in the severe group. Regarding local complications, the incidence of pancreatic necrosis rose significantly with severity, observed in 53.3% of severe cases versus 10% in moderate and none in mild cases (p < 0.0001). Other complications, including acute pancreatic fluid collection (12% overall; 20% in severe), infected necrosis (7% overall; 20% in severe) and pseudo cyst formation (3% overall; 6.7% in severe), were mostly confined to the severe group. Specifically, infected necrosis showed significant differences across severity (p = 0.0033), while pseudocyst formation did not reach statistical significance (p = 0.2681). Systemic complications followed a similar severity-dependent pattern. Acute kidney injury occurred in 11% of patients overall, with 30% in severe and 6.7% in moderate cases (p = 0.0003). Respiratory failure occurred in 10% overall and was present in 26.7% of severe and 6.7% of moderate cases (p = 0.0009). Shock was noted in 16.7% of severe cases only (p = 0.0021) and multiorgan dysfunction was observed in 33.3% of severe and 6.7% of moderate cases (p < 0.0001), with none in the mild group. These findings underscore the prognostic relevance of the Revised Atlanta Classification in predicting both morbidity and mortality in acute pancreatitis. A comparative analysis of key clinical and biochemical parameters between patients who survived and those who died due to acute pancreatitis is presented in Table 4. Patients who succumbed to the disease (n = 5) were significantly older, with a mean age of 62.6 ± 7.09 years, compared to 40.41 ± 13.66 years in survivors (p = 0.0005). Renal dysfunction was markedly worse in non-survivors, evidenced by significantly elevated urea (78.72 ± 66.08 mg/dL) and creatinine (2.69 ± 1.68 mg/dL) levels compared to survivors (24.09 ± 13.99 mg/dL and 0.84 ± 0.43 mg/dL, respectively; both p < 0.0001). Inflammatory burden was also notably higher in the mortality group, with a mean CRP of 182.61 ± 141.55 mg/L and CRP/Albumin ratio of 107.18 ± 106.30, in contrast to 75.98 ± 82.76 mg/L and 28.09 ± 38.10, respectively, in discharged patients (p = 0.0081 and p < 0.0001). Serum albumin levels were significantly lower in non-survivors (2.11 ± 0.87 g/dL) versus survivors (3.20 ± 0.98 g/dL; p = 0.0080). The BISAP score, a validated marker of severity, was dramatically higher in the death group (4.6 ± 0.55) compared to survivors (0.85 ± 0.99; p < 0.0001). Furthermore, the mean hospital stay was longer among non-survivors (7.0 ± 1.9 days) than those who were discharged (5.4 ± 1.7 days; p = 0.0442). These findings, as detailed in Table 4, highlight the prognostic importance of age; renal markers, CRP, albumin, CRP/Albumin ratio and BISAP score in predicting mortality among patients with acute pancreatitis. To evaluate the prognostic accuracy of various clinical and biochemical parameters in predicting mortality in acute pancreatitis, Receiver Operating Characteristic (ROC) curves were generated (Figure 1 - see PDF). The BISAP score exhibited perfect discriminatory ability with an Area under the Curve (AUC) of 1.000, making it the most reliable predictor of mortality in this cohort. Serum creatinine also showed excellent predictive value (AUC = 0.985), followed closely by age (AUC = 0.952) and the CRP/Albumin ratio (CAR) (AUC = 0.930). C-reactive protein (CRP) alone demonstrated good diagnostic performance (AUC = 0.892), while serum albumin had moderate accuracy (AUC = 0.822). Haematocrit, however, yielded a lower AUC (0.622), indicating limited predictive utility. Figure 1 (see PDF) show the ROC curves illustrate the diagnostic performance of various clinical and biochemical parameters in predicting mortality among acute pancreatitis patients. The BISAP score demonstrated the highest predictive accuracy with an AUC of 1.000, followed by serum creatinine (AUC = 0.985) and age (AUC = 0.952). CRP/Albumin ratio, CRP and urea showed moderate predictive ability, while serum albumin had poor discrimination. These findings highlight the utility of BISAP score and renal function markers as reliable prognostic tools in clinical settings.

## Discussion:

This observational study evaluated the clinical, biochemical and radiological predictors of severity and mortality in acute pancreatitis (AP), with a particular focus on the CRP/Albumin ratio (CAR) and BISAP score. Our findings highlight the utility of these tools for early risk stratification, especially in resource-limited settings. Most patients were middle-aged, with the highest incidence observed in the 41- -50-year age group (28%), followed by the 31--40-year age group (23%). A similar age distribution has been reported by Kaplan *et al* and Huang *et al.,* who also noted a predominance of AP among middle-aged adults [7, 8]. Males constituted 64% of the study population, likely reflecting greater exposure to alcohol, smoking and other behavioral risk factors. A large proportion of patients belonged to lower socio-economic strata (72%) and rural backgrounds (57%), which is consistent with observations by Xu *et al.* who associated an increased disease burden in deprived populations with unhealthy lifestyle practices and delayed access to healthcare [9]. Abdominal pain was the most common presenting complaint (100%), followed by vomiting (81%) and abdominal distension (25%). More severe clinical manifestations such as icterus (12%), dyspnea (10%) and altered sensorium (4%) were predominantly seen in severe cases and were associated with higher mortality. Similar findings have been reported previously [10, 11]. Gallstones (35%) and alcohol use (25%) were the most common etiological factors. Combined alcohol and tobacco consumption was present in 15% of patients, while overall tobacco use was noted in 86.6%. Yadav *et al.* and Buxbaum *et al.* demonstrated a strong association between smoking and pancreatitis, while alcohol has also been shown to significantly increase disease risk [12, 13]. Comparable trends were described by Singh *et al.* [14]. Non-survivors were significantly older than survivors (62.6 ± 7.09 years vs. 40.41 ± 13.66 years; p = 0.0005), indicating the adverse prognostic effect of advanced age, likely due to associated comorbidities and reduced physiological reserve [15, 16]. Renal dysfunction emerged as a strong predictor of mortality. Serum urea and creatinine levels were markedly higher in non-survivors (urea: 78.72 ± 66.08 mg/dL; creatinine: 2.69 ± 1.68 mg/dL) compared with survivors (24. 09 ± 13.99 mg/dL and 0.84 ± 0.43 mg/dL, respectively; p < 0.0001). These findings are consistent with established severity scoring systems such as BISAP and APACHE-II, in which renal parameters play an important role [3, 15]. Inflammatory markers were also significantly associated with poor outcomes. CRP levels were substantially higher in non-survivors (182.61 ± 141.55 mg/L vs. 75.98 ± 82.76 mg/L; p = 0.0081). Similarly, CAR was significantly elevated in non-survivors (107.18 ± 106.30 vs. 28.09 ± 38.10; p < 0.0001), suggesting that a combination of systemic inflammation and poor nutritional status predicts severe disease and mortality. Similar observations have been reported in previous studies evaluating CAR in acute pancreatitis [15, 17, 18]. In a prospective study of 150 Indian patients, Yogesh *et al.* demonstrated that the CRP/Alb ratio calculated within 24 hours reliably predicted persistent organ dysfunction, with a cut-off of 0.25 showing 85% sensitivity, 80% specificity, and an AUROC of 0.82, which was significantly higher than CRP alone (AUROC 0.72, p = 0.03) and the Ranson score (AUROC 0. 76, p = 0.04); on multivariate regression, CAR >0.25 independently predicted severe AP with an adjusted OR of 5.2 (95% CI 2.8--9.6) [17]. Zhao *et al.,* in a retrospective cohort of 284 patients, further reported that the CRP/ALB ratio on day 2 was associated with severe AP (OR: 1.74, 95% CI: 1.32--2.29), death (OR: 1.73, 95% CI: 1.24--2.41), pancreatic necrosis (OR: 1.28, 95% CI: 1.08--1.50), and organ failure (OR: 1.43, 95% CI: 1.18--1.73), with the day 3 ratio showing an AUC of 0.943 for mortality, surpassing MCTSI (AUC 0.871), supporting CAR as a dynamic, reproducible, non-invasive prognostic tool that can supplement established scoring systems [18]. Serum albumin levels were significantly lower among non-survivors (2.11 ± 0.87 g/dL vs. 3.20 ± 0.98 g/dL), supporting hypoalbuminemia as a marker of poor prognosis [19]. A BISAP score of ≥2 was strongly associated with worse outcomes, including mortality and the requirement for intensive care. This finding is in agreement with previous validation studies of the BISAP scoring system [3, 15, 20, 21]. The length of hospital stay was longer among non- survivors (7.0 ± 1.9 days vs. 5.4 ± 1.7 days; p = 0.0442), reflecting greater disease severity, complications and resource utilization. Although hematocrit values were higher in non-survivors, the difference was not statistically significant (p = 0.4311), suggesting that static hematocrit values may be less reliable than dynamic inflammatory and organ dysfunction markers such as CRP, creatinine, CAR and BISAP score [15]. Receiver operating characteristic (ROC) analysis further supported these findings. BISAP had the highest discriminatory ability for mortality (AUC = 1.000), followed by creatinine (AUC = 0.985), age (AUC = 0.952) and CAR (AUC = 0.930). CRP alone also demonstrated good predictive accuracy (AUC = 0.892), whereas hematocrit had lower predictive value (AUC = 0.622). These findings are comparable to those reported by Mounzer *et al.,* who demonstrated superior performance of validated scoring systems over isolated laboratory markers [15].

## Conclusion:

We show that key clinical and biochemical parameters notably advanced age, elevated urea and creatinine, increased CRP and CRP/Albumin ratio, hypoalbuminemia and a high BISAP score are significant predictors of mortality in acute pancreatitis. Our findings also highlight the prognostic utility of the CRP/Albumin ratio as a simple, cost-effective biomarker that integrates both inflammatory status and nutritional reserve. Implementing these parameters alongside established severity scores such as BISAP can facilitate early risk stratification, optimize patient management and potentially improve clinical outcomes in acute pancreatitis, especially in resource-limited settings.

## Advancement of knowledge: 

This study adds to existing knowledge by demonstrating that the CRP/Albumin ratio (CAR) is a simple, cost-effective and easily available biomarker for early prediction of severity and mortality in acute pancreatitis. It provides valuable evidence from a resource-limited setting, highlighting the practical utility of routinely available laboratory parameters for risk stratification. The study also confirms that the BISAP score remains the most accurate prognostic tool among the parameters assessed. Importantly, it shows that combining CAR with BISAP can enhance early clinical decision-making and improve patient outcomes. Overall, these findings contribute clinically relevant and applicable insights for better management of acute pancreatitis

## Figures and Tables

**Table 1:** Demographic, socioeconomic and etiological profile of patients with acute pancreatitis

**S.N.**	**Parameter**	**Subcategory**	**n**	**Percentage (%)**
1	Age (years)	Mean± SD	-	41.86± 14.67
	Age group	< 20	4	4%
		21-30	16	16%
		31-40	23	23%
		41-50	28	28%
		51-60	20	20%
		> 60	9	9%
2	Sex	Male	64	64%
		Female	36	36%
3	Socioeconomic status	Lower	72	72%
		Middle	26	26%
		Upper	2	2%
4	Residence	Rural	57	57%
		Urban	43	43%
5	Etiology	Gallstones	35	35%
		Alcohol	25	25%
		Alcohol + Tobacco	15	15%
		Hypertriglyceridemia	10	10%
		Idiopathic	10	10%
		Drug-induced	5	5%
6	Outcome	Discharged	95	95%

**Table 2:** Clinical presentation and symptoms of patients with acute pancreatitis

**Symptom / Sign**	**Number of Patients (n)**	**Percentage (%)**
Abdominal pain	100	100%
Nausea/Vomiting	81	81%
Abdominal distension	25	25%
Fever	22	22%
Icterus (Jaundice)	12	12%
Dyspnoea	10	10%
Altered sensorium	4	4%
Shock at presentation	3	3%

**Table 3:** Distribution of clinical outcomes and complications according to severity of acute Pancreatitis (Revised Atlanta classification)

**Outcome/Complication**	**Mild (n=40)**	**Moderate (n=30)**	**Severe (n=30)**	**Total (n=100)**	**P value**
Hospital Stay (days)	5.2 ± 1.1	8.4 ± 2.0	13.1 ± 3.5	8.8 ± 3.9	p<0.0001
ICU Admission	0 (0%)	3 (10%)	20 (66.7%)	23 (23%)	p<0.0001
Mechanical Ventilation Required	0 (0%)	2 (6.7%)	11 (36.7%)	13 (13%)	p<0.0001
Discharged	40 (100%)	29 (96.7%)	26 (86.7%)	95 (95%)	<0.05
Mortality (Death)	0 (0%)	1 (3.3%)	4 (13.3%)	5 (5%)	p=0.0357
Local Complications					
Acute Pancreatic Fluid Collection	2 (5%)	4 (13.3%)	6 (20%)	12 (12%)	0.155
Pancreatic Necrosis	0 (0%)	3 (10%)	16 (53.3%)	19 (19%)	p<0.0001
Pseudocyst	0 (0%)	1 (3.3%)	2 (6.7%)	3 (3%)	0.268
Infected Necrosis	0 (0%)	1 (3.3%)	6 (20%)	7 (7%)	p=0.0033
Systemic Complications					
Acute Kidney Injury (AKI)	0 (0%)	2 (6.7%)	9 (30%)	11 (11%)	p=0.0003
Respiratory Failure (ARDS)	0 (0%)	2 (6.7%)	8 (26.7%)	10 (10%)	p=0.0009
Shock	0 (0%)	0 (0%)	5 (16.7%)	5 (5%)	p=0.0021
Multiorgan Dysfunction Syndrome	0 (0%)	2 (6.7%)	10 (33.3%)	12 (12%)	p<0.0001

**Table 4:** Comparison of clinical and laboratory parameters between discharged and deceased patients

**Parameter**	**Discharged (N=95)Mean ± SD**	**Death (N=5)Mean ± SD**	**P-value**
Age (years)	40.41 ± 13.66	62.6 ± 7.09	0.0005
Hematocrit (%)	33.93 ± 9.27	37.29 ± 9.09	0.4311
Urea (mg/dL)	24.09 ± 13.99	78.72 ± 66.08	<0.0001
Creatinine (mg/dL)	0.84 ± 0.43	2.69 ± 1.68	<0.0001
CRP (mg/L)	75.98 ± 82.76	182.61 ± 141.55	0.0081
Albumin (g/dL)	3.20 ± 0.98	2.11 ± 0.87	0.008
CRP/Albumin Ratio	28.09 ± 38.10	107.18 ± 106.30	<0.0001
BISAP Score	0.85 ± 0.99	4.6 ± 0.55	<0.0001
Hospital Stay (days)	5.4 ± 1.7	7.0 ± 1.9	0.0442
